# Robotic-assisted total knee arthroplasty with MAKO is associated with improved functional outcomes: a systematic review and meta-analysis

**DOI:** 10.1302/2633-1462.611.BJO-2025-0180.R1

**Published:** 2025-11-06

**Authors:** Kabir Sodhi, Jacob Eaton-Brown, Prakrit Raj Kumar, Oluwasemilore Adebayo, Henry K. C. Searle, Andrew J. Metcalfe, Edward T. Davis, Chetan Khatri

**Affiliations:** 1 Warwick Medical School, University of Warwick, Coventry, UK; 2 Department of Trauma and Orthopaedics Surgery, University Hospital Coventry & Warwickshire, Coventry, UK; 3 St George's University Hospitals NHS Foundation Trust, London, UK; 4 The Royal Orthopaedic Hospital NHS Foundation Trust, Birmingham, UK; 5 Institute of Inflammation and Ageing, MRC-Versus Arthritis Centre for Musculoskeletal Ageing Research, University of Birmingham, Birmingham, UK

**Keywords:** MAKO, Total knee arthroplasty, Total knee replacement, Robotic surgery, Patient reported outcome measures, Forgotten joint score, functional outcomes, Robotic-assisted total knee arthroplasty, total knee arthroplasty (TKA), patient-reported outcome measures (PROMs), randomized controlled trials, Western Ontario and McMasters Universities Osteoarthritis Index, Forgotten Joint Score (FJS), Knee Society Scoring, Oxford Knee Score, manual total knee arthroplasty

## Abstract

**Aims:**

To improve functional outcomes following total knee arthroplasty (TKA), robotic systems have been introduced such as the MAKO (Stryker), the most widely used system globally at present. This systematic review aimed to compare the patient-reported outcome measures (PROMs) of robotic TKA (RTKA) to manual TKA (MTKA).

**Methods:**

Five electronic databases were systematically searched for eligible articles that used PROMs to compare MAKO RTKA to MTKA. The primary outcome was the Forgotten Joint Score (FJS). We defined follow-up periods as short (up to three months), medium (three months to one year), and long term (beyond one year). We pooled outcomes combining the Knee Society Scoring System (KSS), Oxford Knee Score (OKS), and Western Ontario and McMaster Universities Osteoarthritis Index (WOMAC). Meta-analyses were conducted using a random-effects model and reported using mean difference (MD) or standardized mean difference (SMD) and 95% CI.

**Results:**

In total, 22 articles evaluating 3,738 TKAs were included: 1,835 MTKAs and 1,903 RTKAs. The evidence level for most studies were IIa, due to few high-level studies. Using FJS, meta-analysis showed little difference at short-term follow-up (MD 11.49, 95% CI -5.62 to 28.59), but found a difference at medium-term follow-up (MD 5.50, 95% CI 2.19 to 8.81). This was not sustained at long-term follow-up (MD 23.89, 95% CI -16.50 to 64.27). Pooling all PROMs showed no difference in the short term (SMD 0.27, 95% CI -0.05 to 0.59), but results favoured RTKA at medium- (SMD 0.46, 95% CI 0.22 to 0.70) and long-term follow-up (SMD 0.40, 95% CI 0.13 to 0.66).

**Conclusion:**

There are few high-level studies, but based on current data MAKO RTKA may result in improved functional outcomes compared to MTKA. Further randomized controlled trials are required to provide robust data and to assess clinical and cost-effectiveness as well as a wider spectrum of early and late outcomes.

Cite this article: *Bone Jt Open* 2025;6(11):1382–1393.

## Introduction

Total knee arthroplasty (TKA) is a high-volume procedure, with over 100,000 performed annually in the UK, costing approximately £600 million.^1^ This number is expected to rise due to a population that is both ageing and with an increasing prevalence of obesity. TKA is an effective treatment for end-stage arthritis, improving pain, function, and health-related quality of life in most people who undergo the procedure.^2^ However, in the long term, there is variation in outcomes, with around 10% dissatisfied, often due to ongoing pain.^3^

To improve functional outcomes, surgeons have looked towards technology such as robotic TKA (RTKA). The MAKO robot (Stryker, USA) is an image-based system which is being adopted rapidly worldwide, and is the most commonly used robotic system for TKA worldwide.^4^ Early studies have shown reduced iatrogenic periarticular soft-tissue injury, and reduced femoral and tibial bone trauma – this may be the reason for reduced inflammatory response after surgery with MAKO.^5^ This in turn may explain early findings of reduced early postoperative pain and reduced hospital stay.^6^

While these early outcomes may be promising, it is not known whether these will translate to improved medium- or long-term functional outcomes when compared to manual TKA (MTKA). Previous systematic reviews have focused on technical outcomes (e.g. component positioning).^7,8^ When patient-reported outcome measure (PROM) instruments have been studied, a mixture of robotic systems were included, which may dilute the effect of an individual system.^9-11^ We analyzed a single technology and, based on previous studies, hypothesized that MAKO RTKA may result in superior functional outcomes as measured by PROMs compared to manual TKA.

## Methods

### Protocol registration

The study protocol was registered with the Prospective Register of Systematic Reviews (PROSPERO; CRD42025606692). This systematic meta-analysis was conducted and reported in line with the PRISMA guidelines (Supplementary Material).^12^

### Search methodology

A comprehensive search was conducted using five electronic databases: Medline, Embase, Scopus, Web of Science, and the Cochrane Database of Systematic Reviews (inception to 24 September 2024). A summary of the search is in Table I. Medical subject heading and Boolean operator terms are displayed in the Supplementary Material.

**Table I.** Article hits by database.

Database	Hits, n
Medline	828
Embase	1,081
Scopus	957
Web of Science	111
Cochrane Database of Systematic Reviews	148
**All databases**	**3,125**

### Eligibility criteria

Studies were included if they met the following criteria: 1) randomized controlled trials (RCTs), case-control studies, and prospective or retrospective cohort studies investigating either unilateral or bilateral primary total knee arthroplasty comparing MTKA to RTKA (using the MAKO system); 2) English full-text manuscript with available data; and 3) studies that published one of the following PROMs: Forgotten Joint Score (FJS),^13^ Oxford Knee Score (OKS),^14,15^ Knee Society Score (KSS),^16^ Western Ontario and McMasters Universities Osteoarthritis Index (WOMAC), and associated subscales.^17^ Studies were excluded if they were 1) non-comparative or did not report the pre-defined outcomes; 2) review articles, case series, and reports; 3) preclinical or animal studies; 4) non-English language studies; or 5) non peer-reviewed/unpublished manuscripts.

We defined the FJS as the primary outcome measure. It is a PROM instrument developed specifically to evaluate function after arthroplasty and is not limited by ceiling effects seen in other instruments.^18,19^

### Study characteristics

**Fig. 1:**
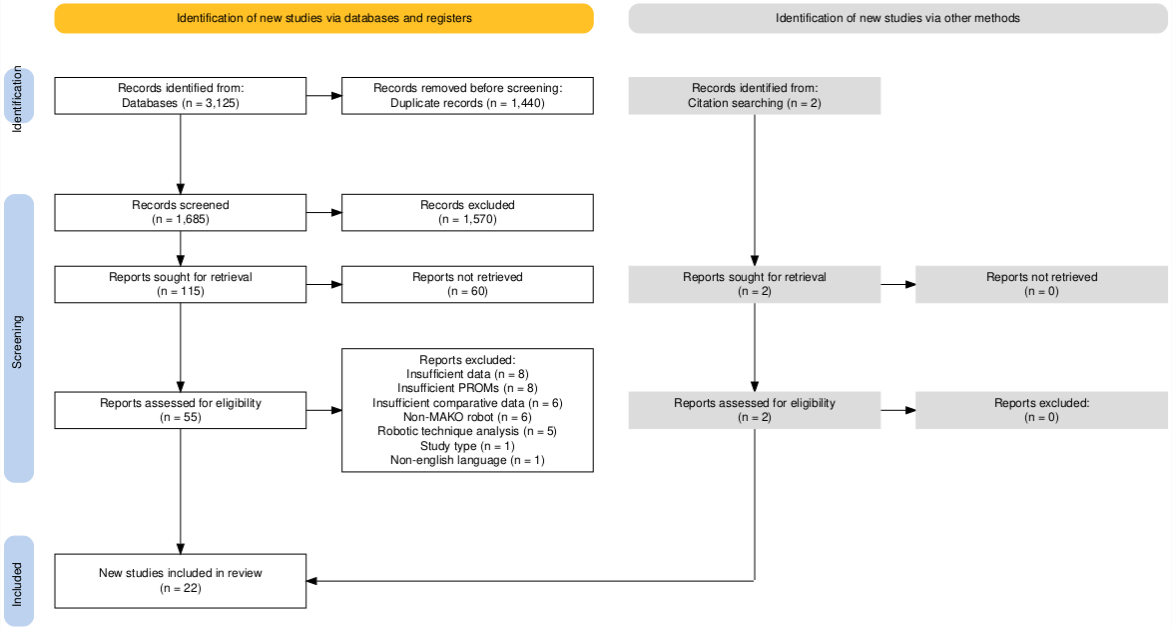
PRISMA flow diagram of study selection. PROMs, patient-reported outcome measures.

The search returned 3,125 potentially eligible articles, and after duplicates were removed, 1,685 articles were identified for screening. After titles and abstracts were screened, 115 studies were eligible for full-text review. In total, 95 were excluded, resulting in the inclusion of 20 studies. A comprehensive examination of full texts of eligible studies led to an addition of two further studies from bibliographical searching. A total of 22 studies were found to meet all inclusion criteria and data requirements out of which 20 were cohort studies and two RCTs (Figure 1) (Table II). The study follow-up period ranged from six weeks to five years. All studies included in this review except Khlopas et al,^20^ Mahoney et al,^21^ McCormick et al,^22^ and Marchand et al^23^ matched their study groups in terms of participant age. All studies included in this review except Smith et al^24^ matched their study groups in terms of BMI. All studies in this review except Boucher et al,^25^ Kafelov et al,^26^ Mahoney et al,^21^ and Zhang et al^27^ matched their study groups in terms of sex. BMI was not reported in Marchand et al^28,29^ (Supplementary Figures a to c).^21-37^ In total, 1,835 MTKAs and 1,903 RTKAs were analyzed, a total of 3,738 TKAs overall.

**Table II.** Characteristics of included studies.

Study ID	Study design	Retrospective vs Prospective	Level of evidence	Number of knees	
				rTKA (n = 1,903)	mTKA (n = 1,835)
Ali et al 2023^38^	Cohort	Retrospective	3	36	36
Boucher et al 2022^25^	Cohort	Retrospective	3	102	137
Choi et al 2023^31^	Cohort	Prospective	3	60	60
Clement et al 2023^34^	RCT	-	2	46	41
Clement et al 2024^39^	RCT	-	2	*	*
Kafelov et al 2023^26^	Cohort	Retrospective	3	100	100
Kang et al 2024^40^	Cohort	Retrospective	3	79	61
Kayani et al 2023^5^	Cohort	Prospective	2	60	60
Khlopas et al 2020^20^	Cohort	Prospective	2	150	102
Marchand et al 2023a^28^	Cohort	Retrospective	3	80	80
Lee et al 2024^35^	Cohort	Retrospective	3	70	70
Li et al 2024^36^	Cohort	Retrospective	3	133	197
Mahoney et al 2022^21^	Cohort	Prospective	2	143	86
McCormick et al 2023^22^	Cohort	Retrospective	3	186	164
Mulpur et al 2022^37^	Cohort	Prospective	3	55	55
Naziri et al 2019^32^	Cohort	Prospective	2	40	40
Marchand et al 2017^30^	Cohort	Prospective	2	20	20
Marchand et al 2019^23^	Cohort	Retrospective	3	53	53
Marchand et al 2023b^29^	Cohort	Prospective	2	210	210
Smith et al 2021^24^	Cohort	Prospective	2	120	103
Winnock de Grave et al 2023^33^	Cohort	Prospective	2	40	40
Zhang et al 2022^27^	Cohort	Retrospective	3	120	120

* Clement et al 2024 is an update of Clement et al 2023.^34^

RCT, randomized controlled trial; TKA, total knee arthroplasty.

### Data extraction

Two reviewers (KS, JEB) independently screened articles based on title and abstract, removing duplicates using Rayyan.^41^ Full texts were obtained for potentially eligible studies and were independently assessed using the eligibility criteria. Data were extracted into a pre-templated form that included: surname of first author, study title, year of publication, study design, number of participants, mean age in years, sex, BMI, length of follow-up, and PROM instruments mean and SD.

It was anticipated that studies would report outcomes at various timelines. Therefore, outcomes were grouped according to their timing: short term (zero to three months inclusive), medium term (greater than three months to one year inclusive), and long term (greater than one year). Where two studies reported the same cohort at different timepoints, data from the longest-follow up were used to prevent ‘double counting’ of participants; data were extracted for all included timepoints.

### Risk of bias assessment

Two reviewers (KS, JEB) independently assessed risk of bias in the included cohort studies using the Risk Of Bias In Non-Randomized Studies - of Interventions (ROBINS-I) tool.^42^ For RCTs, version 2 of the Cochrane risk-of-bias tool for randomized studies (RoB 2) was used.^43^

### Statistical analysis

Data were converted to mean and SD if presented using median or IQRs.^44-46^ A meta-analysis using a random-effects model was performed comparing the RTKA to MTKA. Mean differences (MD) with 95% CIs were estimated for each individual PROM. To pool studies into one meta-analysis for PROMs measuring function as a construct, standardized mean differences (SMD) with 95% CI were used. If studies reported more than one PROM, one PROM was chosen with the following hierarchy: FJS, OKS, KSS-Function, and WOMAC-Physical function. Heterogeneity was assessed using the chi-squared and I^2^ statistic. A chi-squared p-value of < 0.1 was considered as significant heterogeneity. I^2^ values of 0% to 40% were considered not important; 30% to 60% moderate heterogeneity; 50% to 90% substantial heterogeneity; 75% to 100% considerable heterogeneity. Meta-analyses were conducted using Review Manager (RevMan 5.4.1., Denmark). Begg’s funnel plot was used to visually evaluate potential publication bias.

## Results

### Functional outcomes

Seven studies used the FJS to evaluate patient-reported outcomes at different timepoints ranging from three months to five years. There was little to no difference in the short term (MD 11.49, 95% CI -5.62 to 28.59; three studies, 347 participants; I^2^ = 93%), but a difference in the medium term, favouring RTKA (MD 5.50, 95% CI 2.19 to 8.81; seven studies, 1,101 participants; I^2^ = 83%). There were no studies reported at four or five months, therefore this medium-term follow-up incorporated six months to 12 months.

**Fig. 2:**
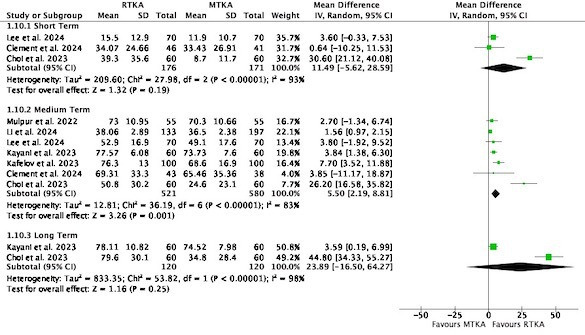
Forgotten Joint Score. df, degrees of freedom; IV, inverse variance; MTKA, manual total knee arthroplasty; RTKA, robotic total knee arthroplasty; TKA, total knee arthroplasty.

This was not maintained at long term (MD 23.89, 95% CI -16.50 to 64.27; two studies, 240 participants; I^2^ = 98%) (Figure 2). It should be noted that there was a paucity of studies, with only two studies at long-term follow-up, one showing a large effect size of RTKA compared to MTKA.

In a sensitivity analysis where analyses were performed according to exact follow-up reported, the FJS was significantly better at one year for RTKA (MD 5.61, 95% CI 2.17 to 9.04; six studies, 542 participants, I^2^ = 86%). At all other timepoints, the FJS was insignificant, although there was a paucity of data at earlier follow-up periods (Supplementary Figure q).

**Fig. 3:**
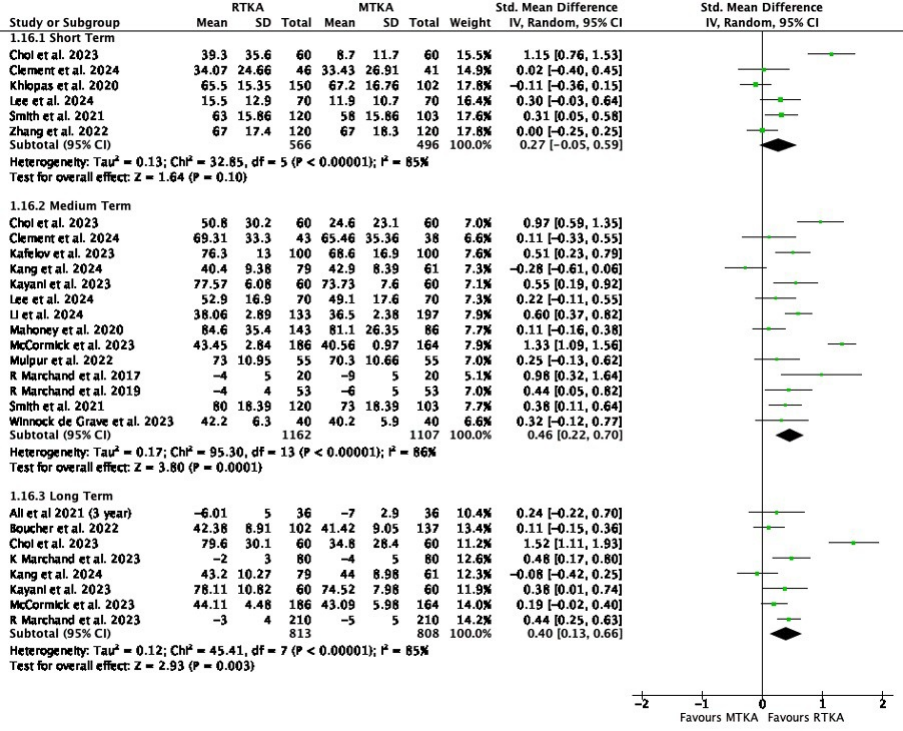
All patient-reported outcome measures. df, degrees of freedom; IV, inverse variance; MTKA, manual total knee arthroplasty; RTKA, robotic total knee arthroplasty; TKA, total knee arthroplasty.

When pooling all studies reporting at least one PROM, there was little to no difference in outcome in the short term (SMD 0.27, 95% CI -0.05 to 0.59; six studies, 1,062 participants; I^2^ = 85%) (Figure 3). There was evidence of a difference in the medium (SMD 0.46, 95% CI 0.22 to 0.70; 14 studies; 2,269 participants; I^2^ = 86%) and long term (SMD 0.40, 95% CI 0.13 to 0.66; eight studies, 1,621 participants; I^2^ = 85%) favouring RTKA. Re-expressing the SMD to FJS showed a MD of 4.84 (95% CI 2.32 to 7.37) in medium term and a MD of 4.21 (95% CI 1.37 to 6.95) in the long term.

In a sensitivity analysis, analyses were performed according to exact follow-up reported. The pooled PROMS favoured RTKA at one year (SMD 2.75, 95% CI 1.45 to 4.04; ten studies, 860 participants, I^2^ = 84%). At all other timepoints the results were insignificant, although there was a paucity of data at earlier follow-up periods (Supplementary Figure r).

Nine studies used at least one section of the KSS to evaluate patient-reported outcomes in RTKA compared to MTKA. Both knee and function scores were evaluated at different time intervals, ranging from the earliest at 0.5 months to the latest at five years.

**Fig. 4:**
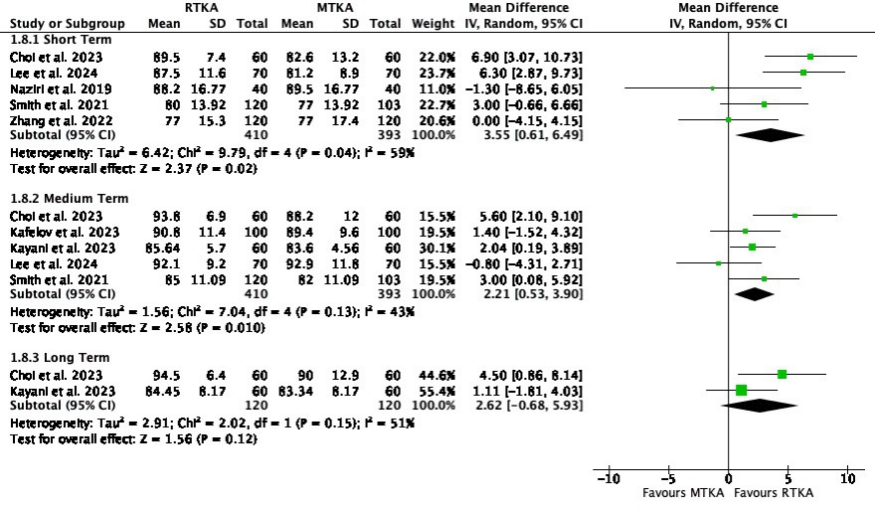
Knee Society Score. df, degrees of freedom; IV, inverse variance; MTKA, manual total knee arthroplasty; RTKA, robotic total knee arthroplasty; TKA, total knee arthroplasty.

Knee scores favoured RTKA in the short term (MD 3.55, 95% CI 0.61 to 6.49; five studies, 803 participants; I^2^ = 59%) and medium term (MD 2.21, 95 CI 0.53 to 3.90; five studies, 803 participants; I^2^ = 43%). However, there was little to no difference in the long term (MD 2.62, 95% CI -0.68 to 5.93; two studies, 240 participants; I^2^ = 51%) (Figure 4).

**Fig. 5:**
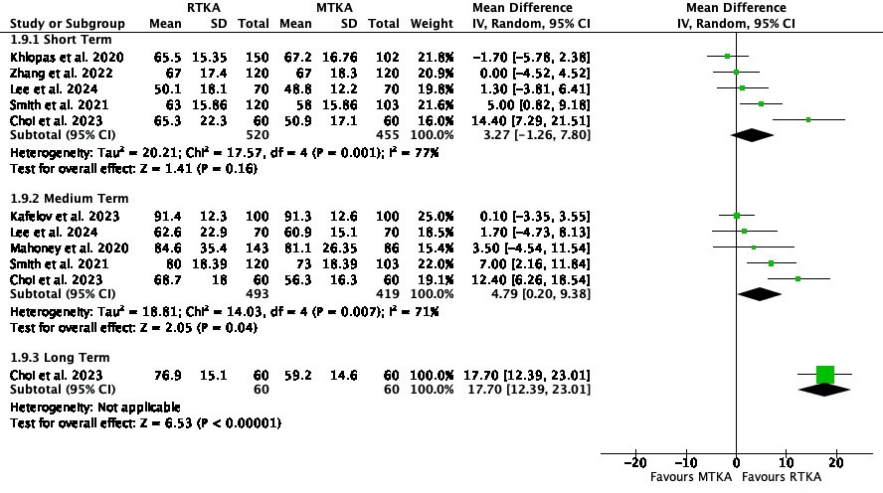
Knee Society Score - Function. df, degrees of freedom; IV, inverse variance; MTKA, manual total knee arthroplasty; RTKA, robotic total knee arthroplasty; TKA, total knee arthroplasty.

Function scores showed little to no difference between scores at short term (MD 3.27, 95% CI -1.26 to 7.80; five studies, 1,475 participants; I^2^ = 77%). However, there was a difference favouring RTKA in the medium term (MD 4.79, 95% CI 0.20 to 9.38; five studies, 912 participants; I^2^ = 71%) and long term (MD 17.70, 95% CI 12.39 to 23.01; one study, 120 participants; I^2^= N/A) (Figure 5).

**Fig. 6:**
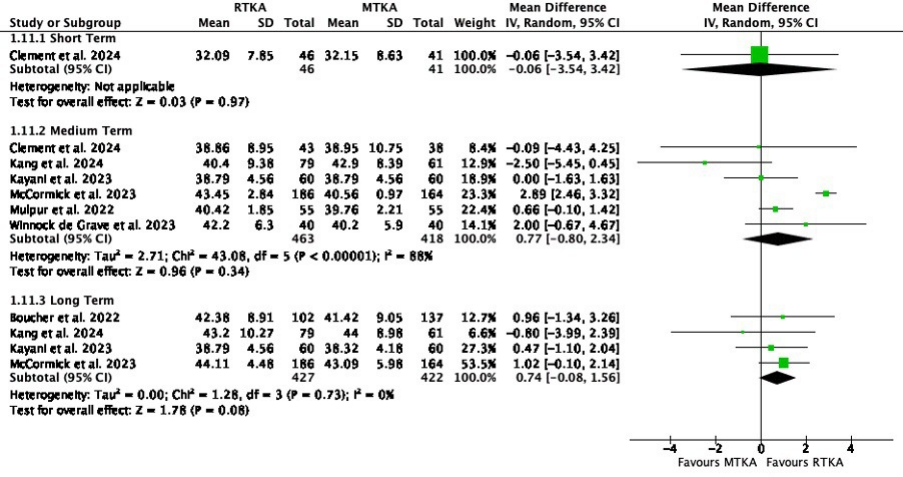
Oxford Knee Score. df, degrees of freedom; IV, inverse variance; MTKA, manual total knee arthroplasty; RTKA, robotic total knee arthroplasty; TKA, total knee arthroplasty.

Six studies used the OKS within a time range of two months to five years. Meta-analysis showed little to no difference in scores at short-term (MD -0.06, 95% CI -3.54 to 3.42; one study, 87 participants; I^2^= N/A), medium-term (MD 0.77, 95% CI -0.80 to 2.34; six studies, 881 participants; I^2^ = 88%) or long-term follow-up (MD 0.74, 95% CI -0.08 to 1.56; four studies, 849 participants; I^2^ = 0%) (Figure 6).

Six studies used the WOMAC. Two studies used the stiffness subscale, while three studies employed the pain and physical function subscales. Some scores used a modified version of WOMAC, and therefore SMD was used for meta-analysis.

**Fig. 7:**
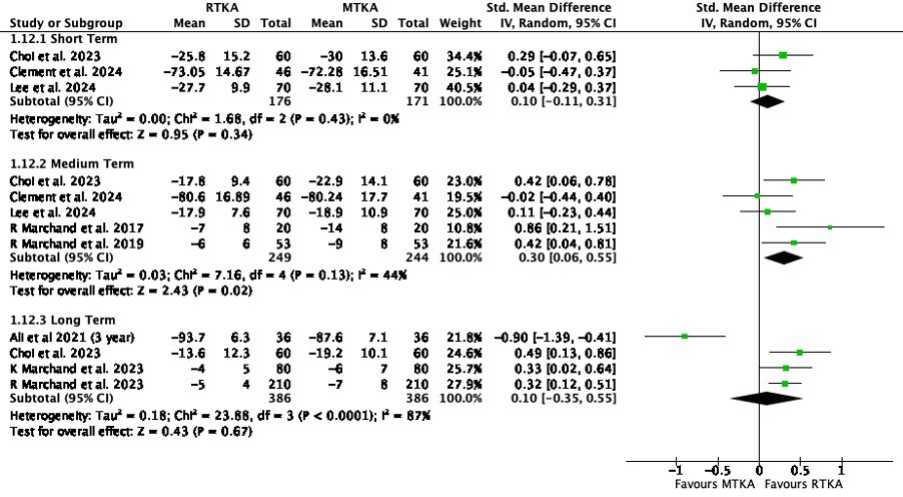
Western Ontario and McMaster Universities Osteoarthritis Index - Total. df, degrees of freedom; IV, inverse variance; MTKA, manual total knee arthroplasty; RTKA, robotic total knee arthroplasty; TKA, total knee arthroplasty.

There was little to no difference in the short term (SMD 0.10, 95% CI -0.11 to 0.31; three studies, 347 participants; I^2^ = 0%), but a difference favouring RTKA in the medium term (SMD 0.30, 95% CI 0.06 to 0.55; five studies, 493 participants; I^2^ = 44%). This was not maintained at long-term follow-up (SMD 0.10, 95% CI -0.35 to 0.55; four studies, 772 participants; I^2^ = 87%) (Figure 7).

**Fig. 8:**
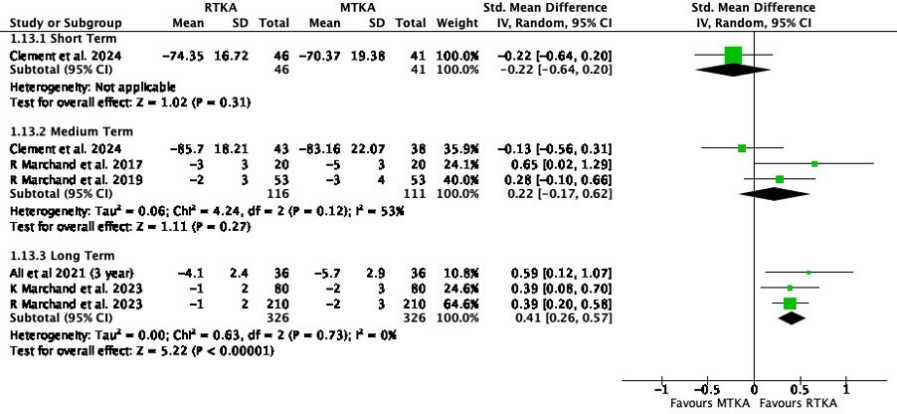
Western Ontario and McMaster Universities Osteoarthritis Index - Pain. df, degrees of freedom; IV, inverse variance; MTKA, manual total knee arthroplasty; RTKA, robotic total knee arthroplasty; TKA, total knee arthroplasty.

In the short (SMD -0.22, 95% CI -0.64 to 0.20; one study, 87 participants; I^2^ = 0%) and medium term (SMD 0.22, 95% CI -0.17 to 0.62; three studies, 227 participants; I^2^ = 53%), there was little to no difference in scores, but a difference was found in the long term (SMD 0.41, 95 CI 0.26 to 0.57; three studies, 652 participants; I^2^ = 0%) (Figure 8).

**Fig. 9:**
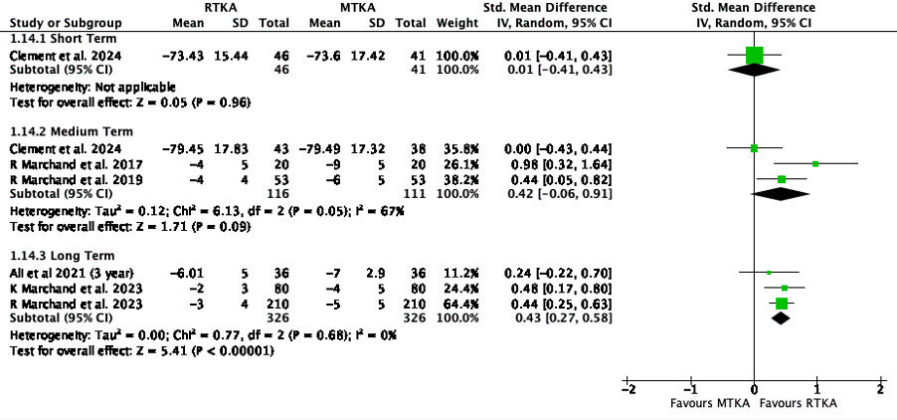
Western Ontario and McMaster Universities Osteoarthritis Index - Physical function. df, degrees of freedom; IV, inverse variance; MTKA, manual total knee arthroplasty; RTKA, robotic total knee arthroplasty; TKA, total knee arthroplasty.

A similar pattern was seen in the WOMAC physical function with little to no difference in the short term (SMD 0.01, 95% CI -0.41 to 0.43; one study, 87 participants; I^2^= N/A) or medium term (SMD 0.42, 95% CI -0.06 to 0.91; three studies, 227 participants; I^2^ = 67%), but a difference in the long term (SMD 0.43, 95% CI 0.27 to 0.58; three studies 652 participants; I^2^ = 0%) (Figure 9).

**Fig. 10:**
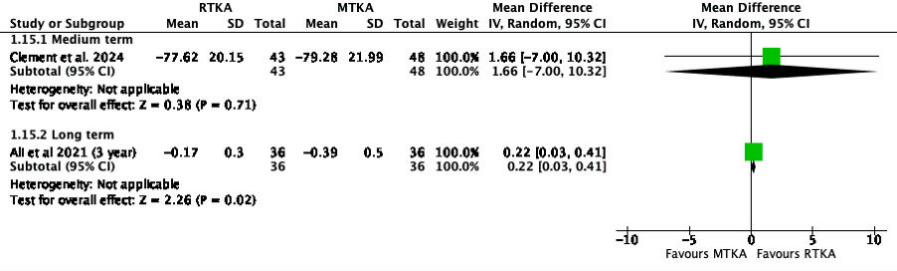
Western Ontario and McMaster Universities Osteoarthritis Index - Stiffness. df, degrees of freedom; IV, inverse variance; MTKA, manual total knee arthroplasty; RTKA, robotic total knee arthroplasty; TKA, total knee arthroplasty.

No studies reported WOMAC-Stiffness in the short term. There was little to no difference in scores in the medium term (MD 1.66, 95% CI -7.00 to 10.32; one study, 91 participants; ; I^2^= N/A) and long term (MD 0.22, 95% CI 0.03 to 0.41; one study, 72 participants; I^2^= N/A) (Figure 10).

### Alignment strategy and level of constraint

Alignment strategy and level of constraint were not consistently reported across studies. Most studies used cruciate-retaining (CR) implants for both RTKA and MTKA. Li et al,^36^ Kafelov et al,^26^ and Smith et al^24^ all used posterior-stabilized (PS) implants for both surgical techniques. Mulpur et al^37^ only specified PS implants for MTKA. Kayani et al^47^ used cruciate-substituting implants for both techniques. Zhang et al^27^ used CR, PS, and varus-valgus constrained implants for RTKA, and CR or PS for MTKA. Information on level of constraint was unavailable for Choi et al,^31^ Naziri et al,^32^ and Lee et al.^35^

Alignment varied significantly across included studies. Eight studies used mechanical alignment (MA) for both MTKA and RTKA. The ROAM trial used MA for MTKA and restricted kinematic alignment for RTKA.^34^ Kafelov et al^26^ and Choi et al^31^ used MA for MTKA and functional alignment for RTKA.^26^ Smith et al^24^ used MA for RTKA but did not specify for MTKA. The alignment technique was unclear or unspecified for nine studies.

### Sensitivity analysis

We performed a series of sensitivity analyses based on changing statistical models, removing studies at high risk of bias and outlier trials. A summary of the sensitivity analyses is provided in the Supplementary Material.

### Risk of bias assessment

The ROBINS-I tool was used to assess risk of bias across selected cohort studies. Bias was assessed in seven domains as indicated by the ROBINS-I tool. Most studies (15/20, 75%) had a moderate risk of bias, with the rest having a severe risk of bias (Supplementary Figure d). The most common cause of risk of bias was bias in measurement of outcomes, which was indicated in every study (Supplementary Figure e).

The RoB 2 tool was used to assess risk of bias in the selected RCT and its update. Bias was assessed across five domains as outlined in the RoB 2 tool. Both were found to have a ‘low’ risk of bias in three of five domains but had ‘some concerns’ in the overall risk of bias Supplementary Figures f and g).

### Publication bias assessment

Funnel plots were constructed for all scoring systems to evaluate the presence and extent of publication bias among the selected studies. All funnel plots, except for WOMAC pain and physical function scores, displayed a high degree of publication bias. This was evident from the asymmetrical distribution of most data points around the central line, indicating potential selective reporting or missing studies (Supplementary Figures h to p).

## Discussion

This systematic review and meta-analysis comprehensively synthesized five key knee-specific functional PROMs comparing MAKO RTKA and MTKA at a range of timepoints (0.5 months to five years) using the FJS as a primary outcome. We found no difference in the short term (up to three months) and long term (beyond one year), although only two studies were included in long-term follow-up. We found better outcomes for RTKA compared to MTKA in the medium term (three months to one year). When meta-analyzing across all PROMs, we found no difference at short term, however functional outcomes favoured RTKA at medium and long term.

The MAKO robotic arm is controlled by the surgeon, with haptic boundaries to minimize errors in cutting bone or soft-tissues. This reduced soft-tissue dissection and iatrogenic damage may result in a reduced inflammatory response after MAKO RTKA.^5,48^ This may explain why a cohort study from the same group reported improved early pain, a finding which is consistent in the literature.^6,30,34,37^ This did not translate to significantly improved early outcomes in this systematic review, but this could be due to heterogeneity between studies as the point estimate was in the direction of robotics at early timepoints. With WOMAC-Pain, which exclusively asks questions related to pain, a difference was only found in the longer-term outcomes favouring RTKA. Other PROMs may not be directly measuring pain, so further evaluation of early pain using specific instruments, such as a visual analogue scale or use of opioid pain relief, may provide greater granularity as to whether RTKA is associated with improved short-term pain as opposed to differences in function.

Several studies have shown that less than 5% of people have a neutral alignment, so while most MTKA results in ‘neutral’ or mechanical alignment, this may not be the natural position for patients. As such, kinematic or functional alignment strategies have been developed.^49,50^ These execute more individualized approaches, directed by the individual’s periarticular soft-tissue envelope. Functional alignment utilizes an alignment plan that is adjusted by the surgeon intraoperatively using RTKA technology to accurately deliver small changes to the angles of the bony cuts. Some studies in this review compared functional alignment delivered by RTKA to mechanical alignment delivered by MTKA.^26,31,35^ This may account for heterogeneity seen in the meta-analyses which may partly be testing functional alignment and mechanical alignment delivered by robotic and manual methods, respectively. Meta-analyzing functional compared with mechanical alignment is difficult as the definition of functional alignment is not universally agreed.^51^

We used the FJS as our primary outcome measure because it was designed to assess functional outcomes after arthroplasty. Other PROMs such as the WOMAC and OKS were designed to assess a preoperative population with knee osteoarthritis. Hence, they display a ceiling effect which may result in a type II error being observed by the instruments not being sensitive enough to detect change.^18,19^ The FJS has not displayed a ceiling effect and is being used in contemporaneous randomized trials comparing RTKA to MTKA.^19,52^

It was unclear in this review whether learning-phase cases for surgeons using RTKA were included. Kayani et al^53^ defined a learning curve of seven cases measured by operating time and heightened levels of anxiety for the surgical team. There was no learning curve associated with implant placement, limb alignment, posterior condylar offset ratio, and joint line restoration but how this affects functional outcomes is not known. A study looking into the learning curve for six surgeons estimated operating times may only normalize between 11 and 43 cases. Inclusion of learning cases into the studies may provide a confounder.^54^

There was only one randomized controlled study included in this review, the ROAM trial, which used the WOMAC as the primary outcome measure.^34^ The study was limited by not having participants or assessors blinded to the intervention. While the ROAM trial found no difference in PROMs between MTKA and RTKA, when combined into meta-analysis, the WOMAC total score significantly favoured RTKA at medium and long term only.

The cost of RTKA requires consideration, especially before adoption into a publicly funded healthcare system such as the NHS. An analysis in the USA found that RTKA was associated with lower 90-day episode of care cost (USD $18,568 vs USD $20,960, p < 0.001 at 90 days).^55^ This did not include capital or hire costs of the robotic system itself and may not be generalizable to other institutions with varying compensation models. In the context of the NHS, Clement et al^34^ found that RTKA was associated with a significant improvement in health-related quality of life, measured by the EuroQol five-dimension questionnaire at two and six months after RTKA (mean difference 0.283 (95% CI 0.188 to 0.378); p < 0.001, and mean difference 0.240 (95% CI 0.138 to 0.341); p < 0.001, respectively). Therefore, a comprehensive clinical effectiveness analysis weighing costs against measured benefit is likely to be important in evaluating any change to RTKA in the future.

The main strength of this paper was the comparison of a single robotic system (MAKO) which is unique due to the delivery of its preoperative 3D planning through a haptically controlled arm, and may confer benefits not seen in other systems. Although this may not be generalizable to other systems, this approach allowed us to evaluate on the most widely used platform.

The aim was to compare the functional outcomes between RTKA and MTKA, using validated knee-related PROMs. Meta-analysis across different instruments facilitated an up-to-date evaluation of MAKO for TKA.

The overall level of evidence was IIa as a meta-analysis of cohort studies.^56^ This reflects the paucity of RCTs comparing PROMs between MAKO TKA and MTKA, with only two randomized trials identified. There are high-level studies evaluating MAKO unicompartmental knee arthroplasty, but further studies are required to assess the value of RTKA.^57^

We captured functional outcomes up to five years in this review. However, further outcomes such as implant survival were not assessed. Future evaluation using registry data will be important to establish the long-term cost-effectiveness of RTKA.

This review meta-analyzed functional outcomes across multiple studies, however it has not meta-analyzed other outcomes such as including early pain and mobility, technical outcomes including component alignment and implant survival, and cost-effectiveness – including health-related quality of life, length of stay, and operating time. RTKA may be associated with fixed capital costs for robotic systems, with additional costs per case for CT scanning and disposable instrumentation.^58^ These issues may be addressed by future reviews. The ongoing RACER-Knee trial compares MAKO RTKA with MTKA, using the FJS as the primary outcome and includes a cost-effectiveness and surgical learning curve analysis. Results are expected in 2025 and could be combined with data from this review.

In conclusion, this systematic review and meta-analysis compared MAKO-assisted robotic TKA to manual TKA and found that RTKA offered superior functional outcomes at medium-term follow-up, as measured by multiple PROM instruments. Using the Forgotten Joint Score, MAKO-assisted TKA resulted in significantly better functional outcomes compared to manual at medium-term follow-up (greater than three months to one year inclusive). There were no differences in short (zero to three months inclusive) and long-term (beyond one year) outcomes, although there was a paucity of data to analyze. When all available PROM instruments were combined in meta-analysis, MAKO-assisted TKA was associated with significantly improved medium- and long-term functional outcomes. Based on this meta-analysis, MAKO TKA may result in better functional outcomes at certain timepoints than manual TKA; however, given the limitations of the included studies, further evidence is required.

Early data that suggested improved short-term recovery (measured by multiple PROM instruments) and pain (measured by WOMAC-pain score) were not seen in this meta-analysis, although the data are relatively heterogeneous. There is a paucity of high-quality studies at present and future randomized controlled trials will build on this evidence to provide more robust estimates to inform clinicians in years to come.

**Take home message**

- MAKO total knee arthroplasty (TKA) may result in better functional outcomes than manual TKA in the medium term.

- Early evidence of improved short-term results for MAKO TKA were not seen in this meta-analysis. There was limited evidence of improved outcomes in the long term, but there was a paucity of studies.

- Further high-quality studies are required to build on this evidence and establish clinical and cost-effectiveness.

## Data Availability

The data that support the findings for this study are available to other researchers from the corresponding author upon reasonable request.
